# The multi-faceted immune modulatory role of S100A4 in cancer and chronic inflammatory disease

**DOI:** 10.3389/fimmu.2025.1525567

**Published:** 2025-02-26

**Authors:** Thomas Wong, Reece Kang, Kyuson Yun

**Affiliations:** ^1^ Department of Neurology, Houston Methodist Research Institute, Houston, TX, United States; ^2^ College of Medicine, Texas A&M University, Bryan, TX, United States; ^3^ Department of Neurology, Weill Cornell Medical College, New York, NY, United States

**Keywords:** S100A4, FSP-1, immune suppression, cancer, fibrosis, chronic inflammation, macrophages, T cells

## Abstract

S100A4 is a Ca^2+^-binding protein involved in multiple chronic inflammatory and neoplastic conditions. This review focuses on recent advances in the understanding of S100A4 function in immune cells, comparing and contrasting S100A4 regulation of immune responses in cancer and chronic inflammatory diseases. We provide evidence that S100A4 regulation of immune cell function has a profound role in promoting the pathogenesis of cancer and pro-inflammatory conditions. Finally, we discuss relevant future directions to target S100A4 therapeutically in different disease states.

## 1 Introduction

The S100 family of proteins contains at least 24 small, Ca^2+^-binding proteins that perform a wide range of functions throughout the cell (1, 2). Both the structure and function of S100 proteins are regulated by Ca^2+^ binding via elongation factor (EF)-hand domains (1). S100 proteins act as Ca^2+^ sensors that respond quickly to changes in Ca^2+^ levels, leading to conformational changes and protein binding that modulate the activity of target proteins. In humans, S100 family genes primarily cluster on chromosome 1q21.3, with some members distributed across chromosomes 4, 5, 21, and X (3). Expression of S100 proteins is highly regulated by oxidative stress, hypoxia, cytokines, growth factors, and toll-like receptor (TLR) ligands, which highlights their importance in maintaining immune homeostasis (2). Amongst them, S100A4 stands out as a member that is strongly implicated in a wide range of pathologies ranging from cancer to chronic inflammatory conditions.

S100A4 is among the most extensively studied member of this family due to its strong association with inflammation, cancer, and neurodegeneration (4–6). S100A4 is also known as fibroblast specific protein-1 (FSP-1), among other names (7), as a gene highly expressed in breast cancer associated fibroblasts. Obviously, this is a misnomer as S100A4 is expressed in many other cell types. S100A4 expression has been associated with chronic inflammatory conditions such as fibrosis (8), rheumatoid arthritis (RA) (9, 10), allergic asthma (11–13) and dermatitis (14). In the context of cancer, S100A4 has been shown to promote metastasis, the epithelial-mesenchymal transition, and immune suppression (5, 15, 16). While many studies have focused on S100A4 function within cancer cells to promote cancer progression (4, 5) and cancer stem cell function (16, 17), a growing body of evidence indicates that S100A4 signaling serves multifaceted roles in the innate and adaptive immune systems. In other words, S100A4 is important in not only the normal immune response but also the pathogenesis and progression of dysregulated inflammatory conditions.

S100A4 is a highly conserved, 101-amino-acid polypeptide, with a C-terminal canonical EF-hand motif and an N-terminal pseudo-EF-hand motif. Ca^2+^ binding to S100A4 promotes its dimerization, which is dependent on the F72, Y75, F78, and L79 residues in helix IV of the canonical EF motif (18). Additionally, S100A4 can form oligomers, including heterodimers with other S100 proteins (19). The dimeric and oligomeric forms of S100A4 have non-overlapping roles. For example, oligomeric, but not dimeric, S100A4 stimulates neuronal differentiation and neurite outgrowth (20) as well as monocyte activity (21). Conversely, dimeric, but not oligomeric, S100A4 interacts with binding partner myosin-IIA (22).

S100A4 protein has been observed in the nucleus, cytoplasm, and extracellular space, and it functions both intracellularly and extracellularly (6). In the cytoplasm, S100A4 regulates cell motility and invasion by binding to non-muscle myosin IIA, rhotekin, tropomyosin, and F-actin (2, 23–26). In the nucleus, S100A4 binds to the C-terminal transactivation domain of p53 to modulate its pro-apoptotic function and promotes p53 degradation (27). In addition, S100A4 regulates expression of transcription factors that control the proneural-mesenchymal transition in glioma stem cells (16). Extracellularly, S100A4 has cytokine-like function and can promote inflammation, angiogenesis, and extracellular matrix remodeling (6). Externalization of S100A4 is typically associated with stress and pathological conditions, but this mechanism remains elusive because S100A4 lacks a signal peptide for secretion (6). Recent studies show that S100A4 may be released via exosomes (28–30). Another possibility is that S100A4 is partially released from dying cells to promote its pathological effects. For example, a recent study demonstrated that DNA-bound S100A4 was released from dying cancer cells to promote metastatic function in neighboring cancer cells via the RAGE pathway (31).

S100A4 is also considered a damage-associated molecular pattern (DAMP) family member. When outside the cell, S100A4 binds to its receptors RAGE (32), TLR4 (9, 21, 29), Embigin (33), Annexin A2, and IL-10R (34) to activate predominantly pro-inflammatory pathways, such as JAK/STAT, MAPK, and NF-κB. Hence, both intracellular and extracellular S100A4 signaling play major roles in regulation of the immune system. This review focuses on the biology of S100A4 in various immune cell subtypes and examines S100A4 activities in the contexts of immune responses in cancer, autoimmunity, and fibrosis.

## 2 S100A4 function in myeloid cells

### 2.1 Monocytes/macrophages

S100A4 expression affects macrophage function in several different ways. First, S100A4 has a direct role in regulating cell motility via interaction with myosin-IIA (23, 26), and macrophages isolated from *S100a4 -/-* mice have impaired ability to migrate to inflammation sites in a thioglycolate-induced model of peritonitis (23). This impairment is attributed to deficient CSF1R signaling and over-assembly of non-muscle myosin IIA in *S100a4 -/-* macrophages (23). Second, S100A4 can modulate cell invasion, and *S100a4 -/-* macrophages were shown to regulate cell invasion independently of myosin IIA via control of podosome rosettes and microtubule acetylation (24). Finally, S100A4 activates RAGE-dependent NF-κβ signaling in RAW 264.7 macrophages (35).

#### 2.1.1 S100A4 regulates macrophage polarization and immunosuppression in cancer

**Figure 1:**
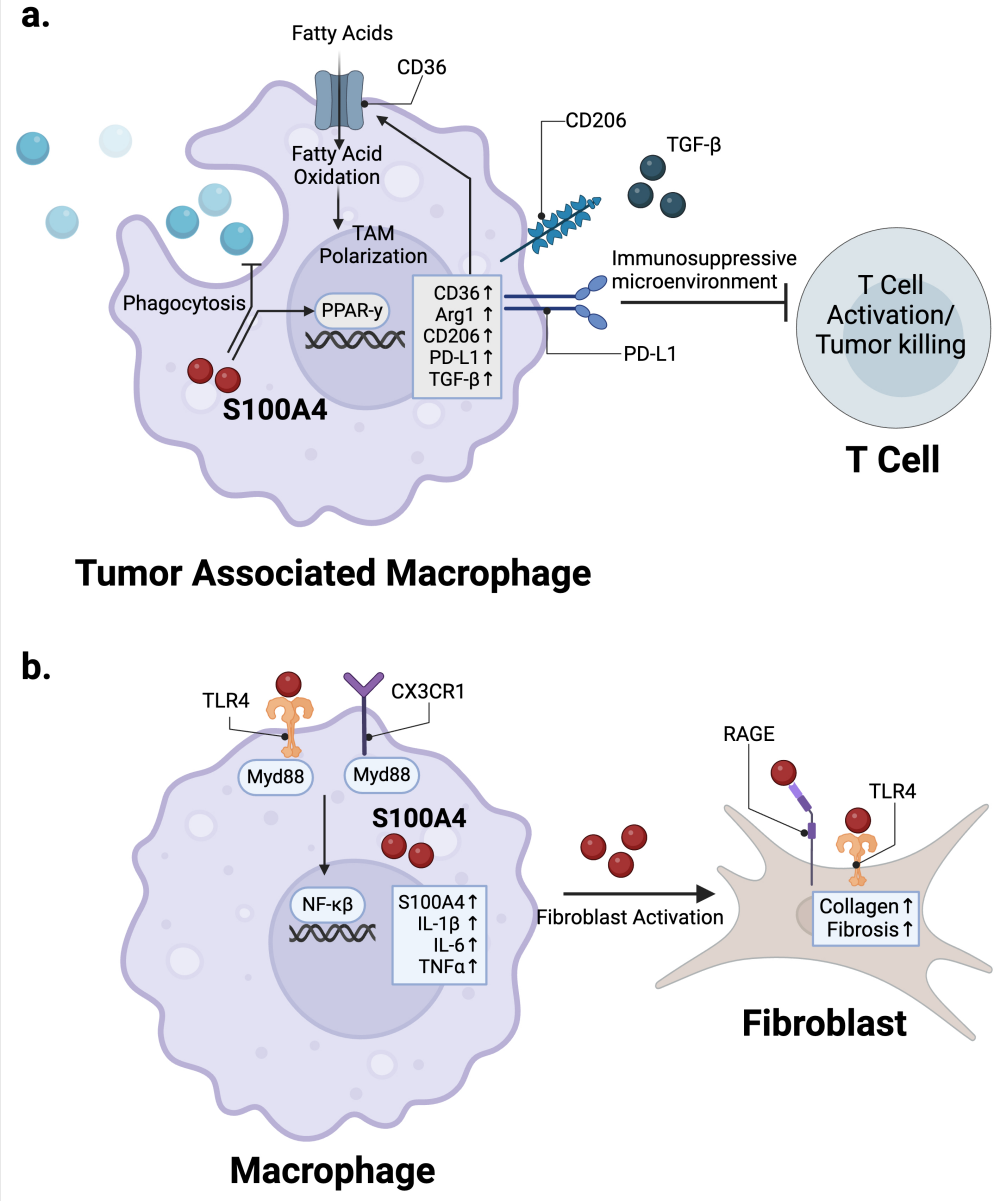
S100A4 regulation of macrophages in cancer and fibrosis. **(a)** S100A4 expression in TAMs activates PPAR-γ signaling to promote expression of immunosuppressive markers such as Arg1, CD206, PD-L1, as well as CD36, which upregulates fatty acid metabolism to promote TAM polarization. **(b)** TLR4 and CX3CR1 signaling in macrophages upregulate S100A4 expression, which then activates fibroblasts via RAGE and TLR4 in the context of fibrosis. Created in BioRender. Wong, T. (2025) https://BioRender.com/r88b394.

In the context of cancer, S100A4 plays an important role in polarizing macrophages towards a pro-tumor, M2-like, alternatively activated state (36). Immunosuppressive, tumor-associated macrophages (TAMs) across human cancer types express high levels of S100A4 (15, 37–39). In TAMs from human liver cancer patients, S100A4 was identified as a key gene associated with macrophage polarization and infiltration (38, 39). In mouse models of glioma, we showed that *S100a4 -/-* TAMs have enhanced phagocytic activity compared with wild-type TAMs (15). Mechanistically, S100A4 expression in macrophages can induce expression of M2-like immunosuppressive surface and functional markers CD206, Arginase-1 (Arg-1), PD-L1, and TGF-β in a PPAR-γ-dependent manner (36) (**Figure 1a**). Additionally, S100A4 promotes fatty acid uptake in macrophages via induction of CD36, a fatty acid transporter (36) (**Figure 1a**). Multiple groups have shown that fatty acid metabolism regulates TAM differentiation and activation (40, 41). S100A4 may likely regulate macrophage polarization towards the M2-like state in concert with CD36-induced fatty acid metabolism (40, 41) (**Figure 1a**).

S100A4 can also play an indirect role in promoting TAM differentiation and activation. To demonstrate this, Prasmickaite et al. conducted *in vitro* studies by co-culturing THP-1 monocytes with human breast cancer cell lines, as well as studies in primary cultures. They demonstrated that extracellular S100A4 promotes secretion of pro-inflammatory cytokines by breast cancer cells, which triggers THP-1 monocyte-to-macrophage differentiation (42). THP-1 TAMs conditioned in S100A4+ breast cancer cell culture media in turn promote mesenchymal transition of breast cancer cells (42). The authors noted that extracellular S100A4 alone also played a role in macrophage differentiation (42). In a separate study, co-transplantation experiments *in vivo* with THP-1 and 4T1 cells showed that *S100a4* WT, but not *S100a4* knockout (KO), THP-1 TAMs promote tumor growth *in vivo* (36).

#### 2.1.2 S100A4 regulates macrophage-mediated fibrosis and chronic inflammation

S100A4 plays a major role in regulating macrophage function in the context of chronic inflammatory conditions such as fibrosis and RA. Multiple studies have shown that S100A4 expression in macrophages has a pro-fibrotic role (43–46). The role for S100A4 in promoting fibrosis has been recently reviewed by others (8). In brief, evidence suggests that S100A4 signaling via RAGE and/or TLR4 activates the TGF-β/SMAD pathway in fibroblasts to promote fibrosis (47, 48) (**Figure 1b**). Accordingly, targeting S100A4 either genetically or therapeutically via antibodies and small molecular inhibitors attenuates fibrosis in multiple mouse fibrosis models and human disease models (46, 49–51). An underappreciated facet of this biology is that the source of S100A4 within fibrotic tissues appears to be primarily from bone marrow-derived or tissue-resident macrophages, not from fibroblasts, in contrast to its common name FSP1 (fibroblast specific protein-1) (43–46) (**Figure 1b**). This has been demonstrated by multiplex immunofluorescence (IF) and flow cytometry analyses in mouse models as well as tissue samples from patients with liver, lung, and kidney fibrosis, where S100A4+ cells co-express macrophage markers such as F4/80, and CD68 (43–45, 50). Studies from mouse models and human liver fibrosis patients indicate that macrophages are the predominant S100A4 expressing population (44, 46, 52). Secreted S100A4 activates hepatic stellate cells, a pericyte-like population of cells with a major role in promoting fibrosis (53, 54), to increase αSMA and collagen production in a c-Myb-dependent manner (46). In mouse models of liver fibrosis, S100A4+ macrophages/Kupffer cells show increased expression of pro-inflammatory cytokines such as TNFα, IL-1β and IL-6, as well as molecules and cytokines classically associated with immunosuppression and the M2-like alternatively activated state, such as IL-10 and COX2 (44) (**Figure 1b**). S100A4 expression in mouse liver macrophages was shown to be driven by the CX3CR1/Myd88/NF-κβ signaling axis, and targeting this pathway alleviated liver fibrosis in a high-fat diet-driven mouse model of metabolic dysfunction-associated fatty liver disease (MAFLD) *in vivo* (55) (**Figure 1b**). Downregulation of METTL14, a methyltransferase decreased in livers of patients with MAFLD and in mouse models of the disease, promotes this pathological S100A4+ macrophage population.

Similarly in lung fibrosis, S100A4 is highly expressed by tissue resident alveolar macrophages and bone-marrow-derived, M2-like macrophages (43, 56). Multiplexed IF in MHV68 and bleomycin induced mouse models of lung fibrosis show that S100A4-positive macrophages co-express the M2-like marker Arg1 (43). In lung samples from human idiopathic pulmonary fibrosis (IPF) patients, S100A4-positive macrophages co-express CD163, another M2-like macrophage marker, suggesting S100A4 expression is in M2-like macrophages (43). Expression of S100A4 in alveolar macrophages was validated by immunohistochemistry staining in a separate cohort of IPF patients (56). Elevated levels of S100A4 in bronchoalveolar lavage fluid have been detected in IPF patients compared to controls, as well as non-specific interstitial pneumonia, hypersensitivity pneumonitis, and sarcoidosis patients (56).

Mouse alveolar macrophages grown *in vitro* and polarized by IL-4, but not by IFN-γ, increase S100A4 expression, which in turn activates lung fibroblasts and likely contributes to disease progression (43). Consistent with this, *S100a4 -/-* mice are protected from lung fibrosis (43, 50), including in a bleomycin-induced lung fibrosis model. In this model, protection from fibrosis was reversed through the adoptive transfer of S100A4 wild type (WT) macrophages in *S100a4 -/-* mice, demonstrating its causal role in this context (50). It is unclear whether S100A4 expression in macrophages regulates its polarization state, as is the case in TAMs, or if it is merely an effector molecule that drives pro-fibrotic states in lung fibroblasts. Nonetheless, S100A4 clearly plays a pathogenic role in these contexts and is a major target for treatment of these conditions.

S100A4 has also been well implicated in RA, with evidence to suggest an immunomodulatory role in monocytes/macrophages in this context (9, 10). Increased S100A4 levels in both plasma and synovial fluid of RA patients is associated with increased disease severity; high S100A4 levels in serum also correspond to poorer treatment response in RA patients (57). S100A4 activates TLR4 signaling in peripheral blood mononuclear cells (PBMCs) isolated from RA patients, leading to increased inflammatory response (9). This study did not identify which cell types are involved in this signaling, but TLR4 is predominantly expressed on cells of myeloid origin, such as monocytes, macrophages, and dendritic cells. Studies in the context of cancer associated myeloid derived suppressor cells (MDSCs) show that S100A4 directly bind to TLR4, suggesting a direct effect in RA as well (58). Furthermore, monocytes from healthy donors exposed to oligomeric S100A4 have enhanced response to lipopolysaccharide (LPS) stimulation, as characterized by increases in IL-1β, IL-6, and TNFα (21).

#### 2.1.3 S100A4 and the interplay between cancer and fibrosis

Notably, there is a growing recognition of the interplay between S100A4, fibrosis, and cancer. Patients with fibrosis are at a higher risk for developing malignancies, and cancer-associated-fibroblasts (CAF) have been extensively studied to elucidate their role in regulating malignant transformation and progression (59–61). S100A4 has previously been implicated in regulation of CAFs (62, 63). S100A4 expressing CAFs secrete VEGF-A and Tenascin-C to promote metastatic colonization of the lung and liver in mouse breast and colorectal cancer models (62). In mouse models of breast cancer metastasis to the lung, macrophage-derived S100A4 promotes formation of the premetastatic niche via activation of lung fibroblasts (64). In human patients and mouse models of hepatocellular carcinoma (HCC), high S100A4 expression positively associates with liver fibrosis and HCC grade and severity (52).

S100A4-expressing macrophages can also be recruited to the liver to induce fibrosis as an adverse outcome in anti-tumor immunotherapy associated toxicity (65). CD137, also known as 4-1BB, is an inducible co-signaling receptor of the TNF receptor superfamily that has been extensively tested as an immunotherapy target in cancer (66–68), but clinical trials with agonistic anti-CD137 antibodies revealed severe dose-dependent hepatotoxicity (69). Studies in mice showed that agonistic anti-CD137 antibody treatment induces liver fibrosis by recruiting macrophages that secrete S100A4 (65). This promote accumulation of pathogenic CD8 T cells in the liver by prolonging their survival via activation of the Akt pathway (65). This phenotype was reversed in *S100a4 -/-* mice and S100A4-thymidine kinase transgenic mice, where S100A4-expressing cells can be depleted with ganciclovir treatment (65). Combination with antibody blockade of S100A4 also reduced liver fibrosis without inhibiting the anti-CD137 antibody anti-tumor response in the MC38 mouse colorectal cancer (CRC) model, suggesting that simultaneously targeting S100A4 may be viable to alleviate immunotherapy-associated liver toxicity in some contexts (65).

When fibrosis occurs within the TME in the context of cancer, S100A4 has immunomodulatory effect. CAFs can impair immune surveillance and reduce the efficacy of anti-tumor immunotherapy through the TGF-β signaling axis (70–73). This has been demonstrated in syngeneic mouse models of non-small cell lung cancer, metastatic colorectal cancer, and metastatic urothelial cancer (71–73). Additionally, CAFs can regulate TAM function to promote immunosuppression. In breast cancer mouse models, fibrosis regulated by TAMs in a TGF-β dependent manner increases arginine consumption and ornithine production, which reprogrammed CD8 T cells to a dysfunctional state, promoting immunosuppression and tumor progression (74). In contrast, direct depleting CAFs to target fibrosis in pancreatic cancer induces immunosuppression through increase accumulation of regulatory T cells (T-regs) that accelerates disease progression, suggesting a protective role of CAFs in some conditions (75). Macrophages are a central hub for the interplay between these pathological processes, and it will be important to decipher whether the functional state of macrophages may be regulated by S100A4 as both a mediator of macrophage polarization and an effector molecule to signal to other cells, such as fibroblasts.

In summary, these data support a working model where, across disease states such as cancer, fibrosis, and chronic inflammation, S100A4 regulates pathogenic macrophage polarization to an M2-like, alternatively activated state with immunosuppressive and profibrotic functions. In addition, S100A4 is an effector molecule that functions as a signal from macrophages to other stromal cells such as fibroblasts and T cells to promote disease progression.

### 2.2 Myeloid derived suppressor cells

MDSCs describe a heterogenous population of immature (MHCII-negative) myeloid cells that are defined functionally by their ability to suppress T cell activity (76). They accumulate in various pathological conditions, particularly in cancer and chronic inflammation, and contribute to disease progression and resistance to cancer treatments (76).

**Figure 2:**
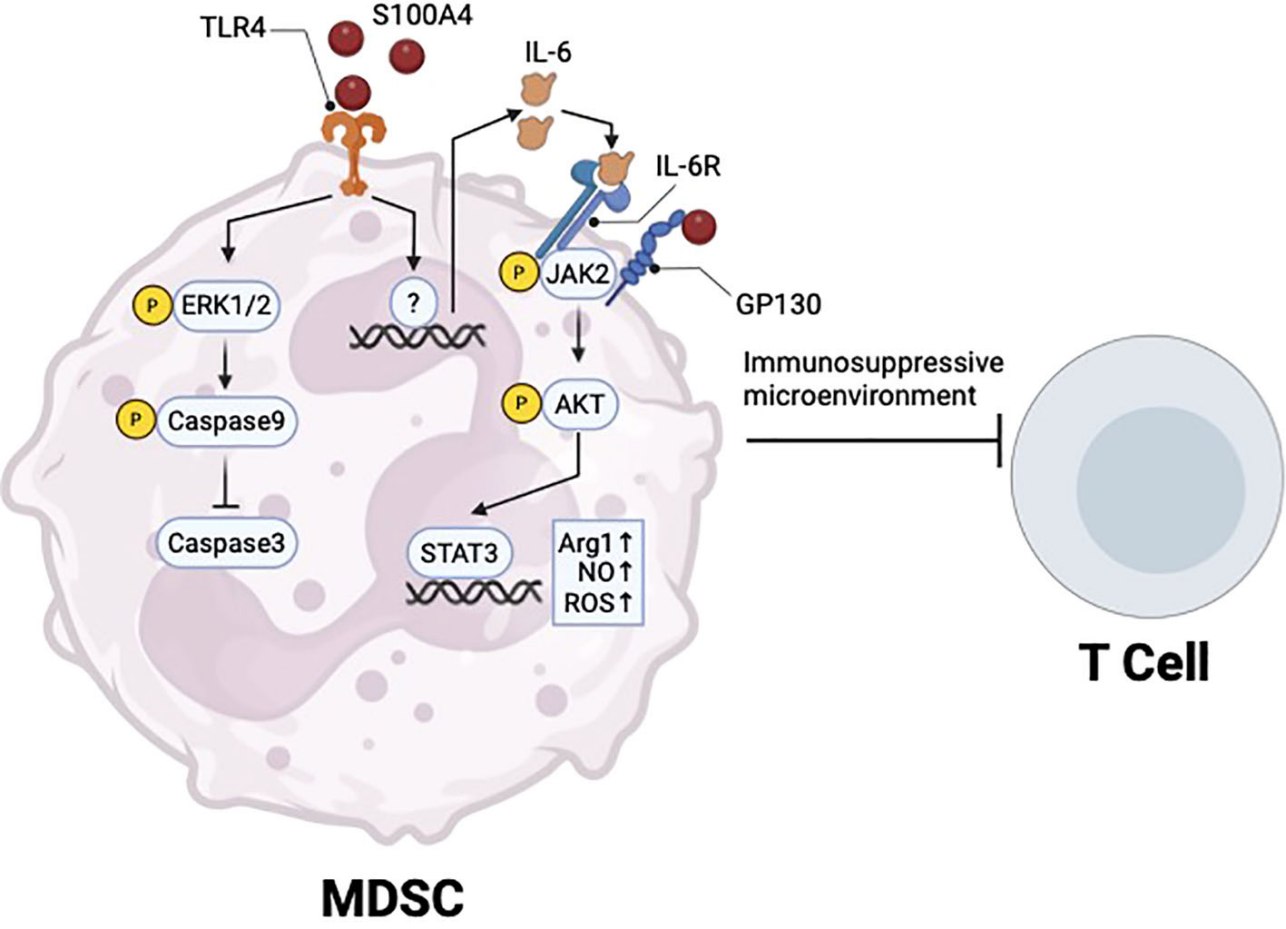
S100A4 regulation of MDSC survival and immunosuppressive function. Extracellular S100A4 binds to TLR4 to promote ERK1/2 signaling and prevent apoptosis, thus promoting MDSC survival in cancer. S100A4-TLR4 axis also upregulates IL-6, which can act through autocrine signaling to activate JAK2/STAT3, which promotes the immunosuppressive function of MDSCs. Created in BioRender. Wong, T. (2025) https://BioRender.com/f68w574.

S100A4 binding to TLR4 regulates MDSCs in different disease states. In cancer, *S100a4 -/-* mice are partially protected from tumor growth due to loss of circulating MDSCs (58). Additionally, exogenous S100A4 protects MDSCs from 5-fluorouracil-induced apoptosis by activating TLR4-ERK1/2 signaling (58) (**Figure 2**). Consistent with this, a different study showed that extracellular S100A4 promotes MDSC expansion and suppression of T cell proliferation and activation in the context of acute myeloid leukemia (AML) (77). This study showed that S100A4 binds to GP130 on MDSCs to activate JAK2/STAT3 signaling (77). GP130 is a newly identified receptor of S100A4, and S100A4 mediated activation of the GP130/JAK2/STAT3 signaling pathway in MDSCs promotes expression of Arg1, TGF-β, and IL-10 (77).

In a separate study of Sjogren’s syndrome, a long-term autoimmune disease, S100A4 signaling via olfactory ecto-mesenchymal stem cell-derived (OE-MSC) exosomes promoted IL-6 production in MDSCs via TLR4 signaling, which led to activation of the JAK2/STAT3 pathway (29) (**Figure 2**). This led to upregulation of Arg1 expression, as well as nitric oxide (NO) and reactive oxygen species (ROS) production in MDSCs, to promote immunosuppressive function (29) (**Figure 2**). *In vitro*, blockade of TLR4 using a neutralizing antibody prevented IL-6 production in MDSCs, while blockade of MDSC-derived exosomal IL-6 by siRNA knockdown reversed immunosuppressive effects on CD4 T cell proliferation (29).

Interestingly, exogenous S100A4 has been shown to promote IL-6 production in monocytes, while S100A4-positive macrophages in the liver were shown to have increased expression of IL-6 transcripts compared with S100A4-negative counterparts. IL-6 is a driver of M2-like polarization in macrophages via activation of JAK2/STAT3 signaling (78, 79). In addition to exogenous S100A4 signaling, single-cell RNA sequencing (scRNAseq) of tumor infiltrating immune cells in glioma patients showed that S100A4 is highly expressed in MDSCs and immunosuppressive macrophages (15). These observations suggest that exogenous and endogenous S100A4 expression is correlated with IL-6 expression in different myeloid cells and may underlie the role of S100A4 in immune suppressive myeloid cells (MDSC and M2-like macrophages) (21, 44).

### 2.3 Dendritic cells

**Figure 3:**
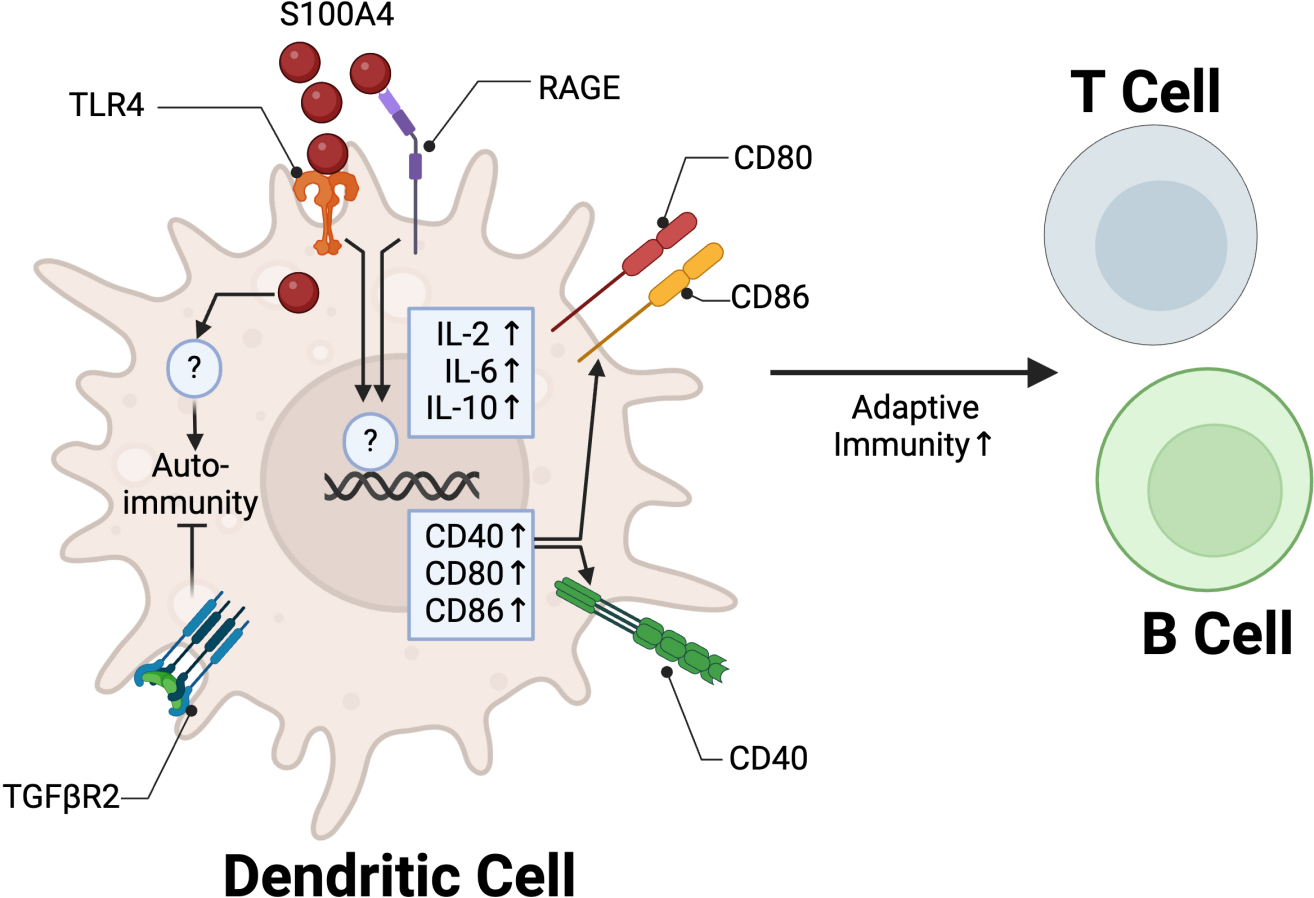
S100A4 regulation of DC function in adaptive immunity. In DCs, extracellular S100A4 binds to TLR4 and RAGE to promote adaptive immunity through upregulation of IL-2 and IL-6 and co-stimulatory molecules CD40, CD80, and CD86. TGFβR2 signaling in S100A4+ DCs is critical to promote tolerance and prevent autoimmune pancreatitis. Created in BioRender. Wong, T. (2025) https://BioRender.com/y63r777.

While most studies investigating the role of S100A4 in immune cells has focused on macrophages and lymphocytes, some reports provide compelling evidence that S100A4 promotes adaptive immunity through DCs. Ovalbumin (OVA) is a model allergen commonly used to induce a Th2 polarized response (80). *S100a4 -/-* mice are protected from OVA-induced allergic dermatitis, characterized by reduced infiltration of eosinophils, T cells, neutrophils, and DCs at the challenge site, coupled with reduced serum OVA-specific antibodies and T cell memory responses (14). DCs isolated from *S100a4 -/-* mice had reduced capacity to stimulate T cell proliferation *in vitro* (14). This phenotype was partially abrogated by treatment with recombinant S100A4, suggesting that extracellular and intracellular S100A4 signaling in DCs may have non-redundant roles in promoting T cell activation/proliferation. A subsequent study confirmed these results, and further showed that adoptive transfer of WT DCs, but not *S100a4 -/-* DCs, into *S100a4 -/-* mice one day prior to immunization with OVA could restore humoral and cellular immune responses, implicating the importance of S100A4 in regulating adaptive immunity through DCs (81). Extracellular S100A4 signaling through RAGE and TLR4 was shown to potentiate activation of DCs by upregulating both expression of cytokines critical to adaptive immunity, such as IL-2 and IL-6, as well as co-stimulatory molecules, such as CD80, CD86, and CD40 *in vitro* (82, 83). S100A4 induced cytokine expression in DCs was partially mediated by NF-κβ signaling, while co-stimulatory molecules CD80 and CD86 were not, indicating that S100A4 acts through multiple downstream signaling pathways to regulate DC function (82) (**Figure 3**).

S100A4 is also involved in the tolerogenic function of DCs. Human monocyte-derived DC’s pulsed with soluble S100A4 were shown to increase CD4+ CD25+ FOXP3+ regulatory T cell proliferation *in vitro* (84). A separate study showed that TGF-β signaling may mediate immune tolerance in S100A4-expressing DCs. Mice with S100A4/FSP1 Cre-mediated conditional knockout of TGFβR2 (Tgfbr2*^fspKO^*) spontaneously develop autoimmune pancreatitis by 6 weeks of age. Adoptive transfer of Tgfbr2*^fspKO^* DCs, but not WT DCs, into 2-week-old syngeneic WT mice similarly led to induction of autoimmune pancreatitis (85) (**Figure 3**). The molecular mechanisms underlying the interplay between S100A4 and TGF-β in regulating DCs remain. These two players have been implicated in regulating fibroblast activity, with S100A4 as a downstream mediator of TGF-β signaling (48). Overall, these findings suggest that S100A4 plays an essential role in the regulation of adaptive immune responses through positive modulation of DC function to promote inflammation and immune tolerance.

## 3 S100A4 function in lymphocytes

S100A4 is expressed in T cells during lineage commitment and memory differentiation (86), as well as in pathological conditions of asthma (12), arthritis (87), chronic infection (88), and cancer (15). A *S100a4* +/GFP mouse model suggested that during a steady state, S100A4 is primarily expressed in CD4 and CD8 memory T cells compared with naïve populations (86). This was also shown in humans, as bulk RNAseq of sorted human CD8 T cells showed that S100A4 expression was absent in naïve T cells but highly expressed in memory T cell subpopulations (89). Similarly, in pathological conditions such as cancer, viral infection, and chronic inflammation, S100A4 is expressed in activated T cells, T-regs, and exhausted CD8 T cells (15, 87, 88).

While S100A4 was shown to regulate macrophage motility and migration, there are mixed reports regarding T cell migration. S100A4 has been shown to be a chemoattractant for T cells in the context of breast cancer lung metastasis (90, 91), though the receptor mediating this interaction is unknown. *S100a4-/-* T cells had no change in migration *in vitro* towards the chemokine CXCL10 (14). Similarly, in mouse models of autoimmune colitis, *S100a4-/-* T cells sufficiently mediated local autoimmunity (86). In a mouse model of allergic dermatitis, CD8 but not CD4 T cell infiltration at the rechallenge site was decreased in *S100a4 -/-* mice compared to WT controls (14). In the context of experimental autoimmune encephalitis (EAE), a mouse model of multiple sclerosis driven by Th1 and Th17 response (92), global depletion of S100A4 is protective against EAE symptoms by decreasing microglia mediated neuroinflammation (93). However, while autoreactive CD4+ T cells in EAE upregulate S100A4 (94), adoptive transfer of *S100a4-/-* T cells are sufficient to drive disease progression into *Rag2 -/-* mice, indicating T cell recruitment to the central nervous system (CNS) and subsequent function is not impaired by loss of endogenous S100A4 signaling (86). It is worth noting that elevated S100A4 levels have been observed in cerebrospinal fluid of MS patients compared to controls (95); the role of S100A4 in the CNS has been reviewed elsewhere (96).

**Figure 4:**
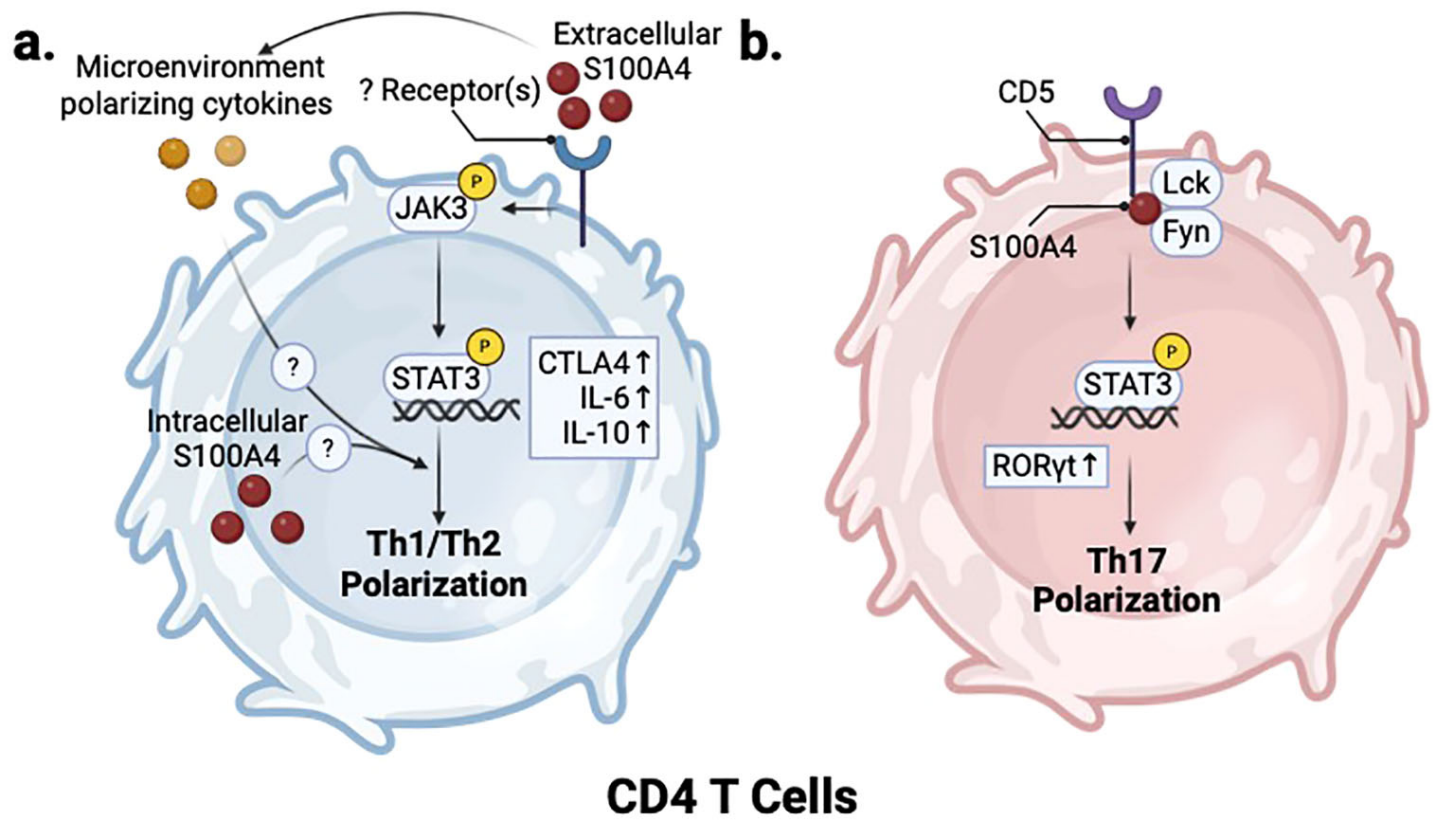
S100A4 regulates T helper cell polarization. **(a)** In CD4 T cells, S100A4-mediated activation of JAK/STAT signaling through an unknown receptor also inhibits Th1 and promotes Th2 polarization. **(b)** In Th17 cells, S100A4 regulates Lck/Fyn activity in CD5 signaling to promote ROR*γ*t expression and induce Th17 polarization. Created in BioRender. Wong, T. (2025) https://BioRender.com/v93n538.

Reports for T cell activation and polarization are conflicting. Treatment of mouse primary T cells with extracellular S100A4 activates JAK/STAT pathway and reduces Th1 polarization while not affecting Th2 polarization, indicated by marker expression and decreased IFNγ and increased IL-10 secretion (90). In line with this, a recent study in mice administered with a *M. tuberculosis* vaccine demonstrated that adjuvant S100A4 protein treatment promotes Th1 and Th17 polarization (82, 83), although it is not clear whether this *in vivo* effect was mediated through direct activation of T cells or through antigen presenting cell (APC) modulation. While extracellular S100A4 appears to partially regulate T cell polarization, multiple studies report that depletion of endogenous S100A4 expression does not impact the capacity of CD4 T cells to differentiation into Th0, Th1, Th2, or Th17 effector cells *in vitro* under polarizing conditions (86). In a study using a model of bacterial infection with ActA-deficient OVA-transgenic *L. Monocytogenes*, *S100a4* -/- mice showed neither deficiency in number of splenic OVA-specific effector CD8 T cells, nor a decrease in IFNγ production after OVA challenge (86). On the other hand, a different study showed that splenocytes from *S100a4 -/-* mice showed decreased IFNγ production post stimulation with an αCD3 antibody, supporting the idea that S100A4 is necessary for robust T cell activation (87). Overall, these studies highlight the context-dependent function of S100A4 in regulating T cell polarization and activation. (**Figure 4a**).

Another study shows that S100A4 also regulates CD8 T cell survival through the Akt pathway (65). Studies with the mouse CD8 lymphoid cell line CTLL-2 showed that extracellular S100A4 treatment *in vitro* significantly reduces apoptosis by IL-2 deprivation. This effect could be reversed with an S100A4 blocking antibody and with an Akt inhibitor (65).

### 3.1 S100A4 regulates T cell function to promote cancer progression and metastasis

S100A4 expression in cancer is highly associated with T cell exhaustion and immunosuppression. Through single cell RNA-sequencing of tissues from glioma patients, we showed that *S100A4* is highly expressed in exhausted, tumor-infiltrating CD8 T cells and T-regs (15). In addition, we showed that S100A4 expression regulates T cell immunomodulatory function in cancer. Using an *S100a4*-GFP knock-in reporter mouse with syngeneic mouse glioma models, we previously showed that GFP+ glioma infiltrating CD4 T cells from *S100a4 -/-* mice had reduced ability to inhibit T cell proliferation *in vitro* compared with their counterparts from *S100a4 GFP/+* mice (15). However, it is unknown if this is due to secretion and extracellular signaling of S100A4 or via intracellular S100A4 signaling. In a separate study with human PBMCs *in vitro*, extracellular S100A4 alone was sufficient to enhance T cell immunosuppression by inducing IL-10 and IDO production in T cells (97). Taken together, these results suggest that both intracellular and extracellular S100A4 have immunomodulatory function in T cells.

**Figure 5:**
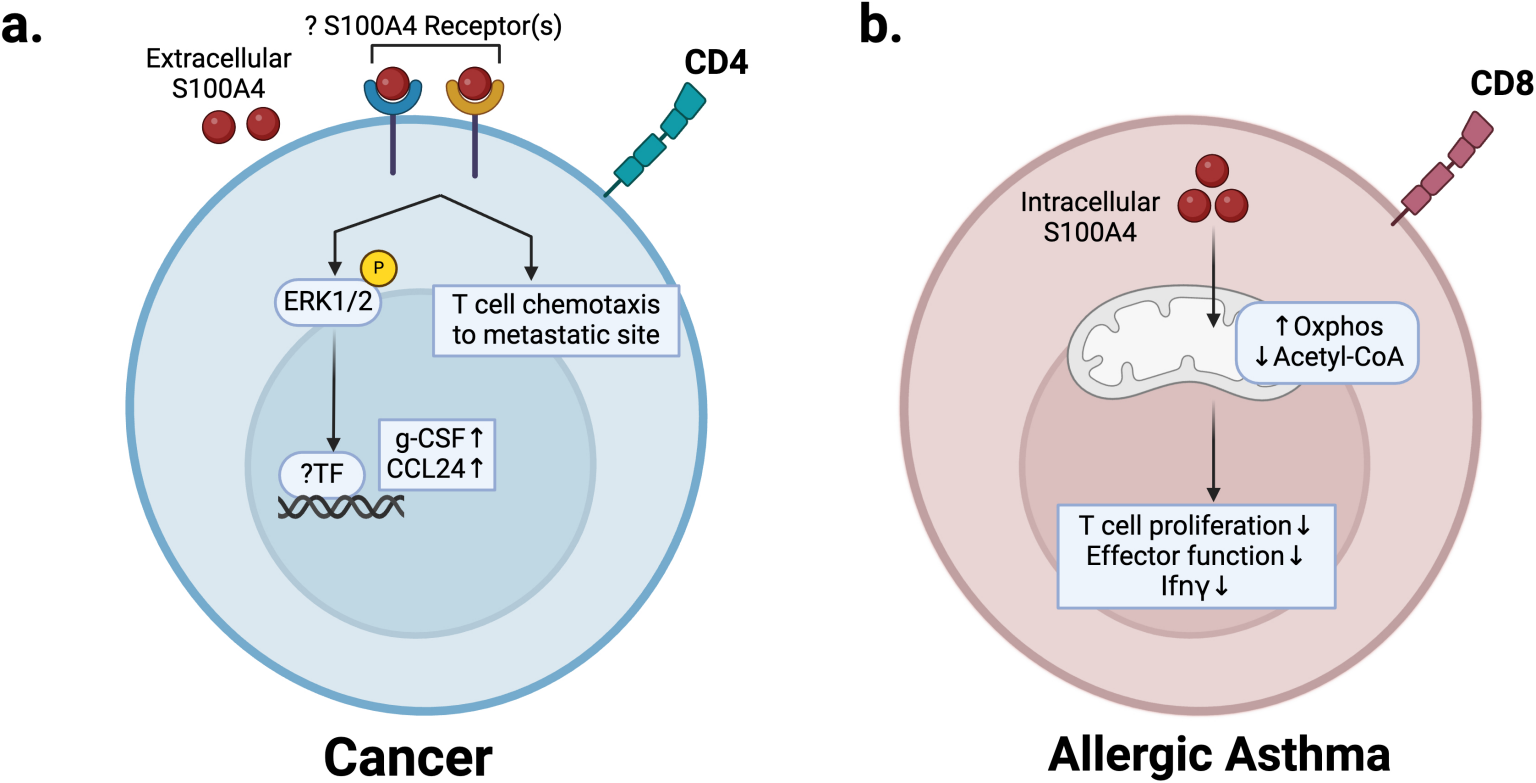
S100A4 regulation of T cells in cancer and allergic asthma disease states. **(a)** In the premetastatic niche, extracellular S100A4 regulates recruitment of T cells and production of pro-inflammatory cytokines g-CSF and CCL24 to promote neutrophil infiltration and metastasis. **(b)** In allergic asthma, S100A4 expression in CD8 T cells promotes mitochondrial metabolism, which reduces acetyl-CoA levels, prevents IFN*γ* production, and decreases effector function, thus promoting pathogenesis. Created in BioRender. Wong, T. (2025) https://BioRender.com/q01y039.

S100A4 can also promote cancer metastasis by enhancing premetastatic niche formation. T cells play a complex role in regulating the pre-metastatic niche, and their pro- or anti-tumor effects are dependent on cell type and functional state (98). CD4 T cells can play a pro-metastatic role by modulating pro-tumor macrophages via IL-4 signaling or by differentiating into immunosuppressive regulatory T cells that inhibit cytotoxic T cell proliferation (99–101). Using the MMTV-PyMT spontaneous metastatic breast cancer mouse model, one study showed that S100A4 secretion by fibroblasts promotes T cell accumulation in pulmonary metastatic sites to promote metastasis to the lung (91). Antibody blockade of S100A4 in the same mouse model also reduced both primary tumor growth and metastatic outgrowth, with reduced T cell recruitment in the pre-metastatic niche (90). Consistently, *S100a4 -/-* mice are protected from both primary tumor growth and metastasis (5, 15, 58, 102). *In vitro* stimulation of T cells with S100A4 revealed increased secretion of cytokines including CCL24 and g-CSF. This was confirmed *in vivo* in interstitial fluid of T cell-infiltrated lungs in tumor-bearing mice (91) (**Figure 5a**). g-CSF is a major regulator of neutrophil production and function (103), and several studies have indicated that immunosuppressive neutrophils promote breast cancer lung metastasis by suppressing cytotoxic T cell function (104–107). Consistent with this, we recently reported that a S100A4 blocking antibody (S100A4-11) treatments inhibits neutrophil infiltration in lungs of breast tumor bearing mice, preventing premetastatic niche formation (108). Notably, we did not observe a significant change in T cell numbers but did observe that TIGIT mediated suppression of T and NK cells is reversed in S100A4-11 treated lungs (108).

### 3.2 S100A4 regulates T cell polarization to promote pathogenesis in chronic inflammatory disease

Allergic asthma is a chronic inflammatory disease of the airways driven by Th2 response and eosinophils (109). S100A4 can regulate the T cell-mediated immune response in allergic asthma (12). S100A4 expression in CD8 T cells is increased in mouse models of allergic asthma and leads to CD8 effector memory T cell dysfunction (12). Mechanistically, S100A4 expression upregulates mitochondrial metabolism in CD8 T cells to decrease acetyl-CoA levels, thus impairing effector gene transcription (**Figure 5b**). This leads to dysfunction of effector memory T cells with high S100A4 expression (S100A4-hi), characterized by impaired IFNγ production (12) (**Figure 5b**). Under conditions of OVA-induced allergic asthma, S100A4-hi CD8 T cells had increased memory development and proliferation compared with S100A4-low-expressing (S100A4-low) counterparts and drove disease progression by inhibiting IFNγ production. S100A4-low, IFNγ-producing CD8 T cells were shown to be protective via adoptive transfer (12). Decreased IFNγ signaling due to apoptosis of S100A4-low IFNγ producing CD8 T cells and persistence of S100A4-hi CD8 T cells drives an increase in Th2 cytokines in the lungs of asthmatic mice, which is reversed in *S100a4 -/-* mice (12).

S100A4 also plays a role in the development of Th17 cells in the context of RA by regulating the CD5-STAT3-IRF4-RORγT axis (**Figure 4b**). An imbalance between FOXP3+ regulatory T cells and RORγT+ Th17 cells promotes the pathogenesis of RA (110). In a methylated bovine serum albumin (mBSA) immunized mouse model of RA, inflamed synovial fluids of *S100a4 -/-* mice showed higher levels of T-regs and lower levels of RORγT+ Th17 cells than their wildtype counterparts. S100A4 deficiency in mice led to a reduced proportion of IL-17α-producing CD4 T cells within the spleen, which correlated with decreased pSTAT3 and RORγT mRNA, two major regulators of Th17 development. In addition, decreased CD5 expression and activity in CD4 T cells were noted in *S100a4 -/-* helper T cells (87). *In vitro* immunoprecipitation experiments using Jurkat cells revealed that S100A4 associates in complex with Src-family kinases Lck and Fyn (87). CD5 was shown to bind directly to the EF2 domain of S100A4 (87) (**Figure 4b**). Importantly, S100A4 modulates CD5 downstream signaling by regulating Lck/Fyn phosphorylation of the CD5 cytoplasmic domain, thus promoting downstream activation of STAT3 to promote Th17 lineage differentiation (87) (**Figure 4b**). CD5 is an inhibitory receptor that downregulates TCR signaling on T cells, and plays a role in Th17 development by promoting IL23 receptor expression and STAT3 activation (111). The purported interaction between S100A4 and CD5 may have implications in other disease contexts, such as cancer, given the ability of CD5 to downregulate T cell response (112, 113).

In line with this, studies using mouse models of chronic viral infection show that S100A4 expression is upregulated in exhausted CD8 T cells (88, 89), suggesting that S100A4 expression may be a signature of exhausted CD8 T cells across different pathological conditions.

In conclusion, these results highlight how S100A4 expression in T cells can polarize T cells into pathogenesis-promoting states, consistent across chronic-inflammatory diseases and cancer.

## 4 S100A4 in immune modulation of cancer

To this point, we have focused on the roles of S100A4 in regulating different immune cell types in normal and pathological contexts such as cancer. However, it is important to also consider that S100A4 is expressed in multiple cell types, including in cancer cells. Secretion of S100A4 from any of these cells can affect multiple downstream cell types including immune cells, thereby impacting the overall immune response. Thus, it is important to understand the overarching immunomodulatory impact of S100A4 within the tumor microenvironment (TME), which has important implications for immunomodulatory therapies. Many studies have focused on S100A4’s ability to promote the progression of cancer and metastasis through regulation of cancer cell motility, cancer cell survival, angiogenesis, cancer stem cell stemness, the epithelial-to-mesenchymal transition, and formation of the premetastatic niche (5, 16, 31, 114–116); however, a growing body of research also implicates S100A4 in immunomodulation of the TME to promote a pro-tumor response (15, 35, 36, 52, 58, 65, 91, 117).

Across cancers, S100A4 expression is associated with poor survival and has a negative impact on the anti-tumor immune response (15, 38, 39, 118), but the mechanism by which S100A4 modulates this response may vary in different cancers. In gliomas, which are characterized by limited T cell infiltration and abundance of immunosuppressive myeloid cells that contribute to therapy resistance (119), depleting S100A4 in stromal cells is sufficient to reprogram the immune microenvironment and increase CD4 and CD8 T cell infiltration, enhance macrophage phagocytosis, and result in significant survival extension (15). In HCC, depleting S100A4 in stromal cells reduces tumor stemness properties and inflammation-associated pathological features (52, 120). Like in liver fibrosis, the majority of S100A4-expressing stromal cells in HCC are of myeloid origin. In colitis-associated CRC mouse models, S100A4 genetic depletion in stromal cells or antibody blockade reduces CRC tumorigenesis (35). This reduction in CRC tumorigenesis is mediated by reduced macrophage and neutrophil infiltration in the colon, resulting in reduced inflammatory response. Decreased S100A4/RAGE-dependent NF-κβ signaling in macrophages also mediates this inflammatory response (35). In mouse models of breast cancer, S100A4 promotes the recruitment of pro-tumor Th2 polarized CD4 T cells to both the primary tumor and metastatic sites to promote disease progression (91, 108, 121), and antibody blockade of S100A4 reduces CD3+ T cell infiltration in the primary tumor and lung metastasis (90). Similarly, T cell accumulation was also reduced by genetic depletion of *S100a4* in stromal cells in MMTV-PyMT and CSML100 tumor bearing mice (90, 91). In a syngeneic mouse model of prostate cancer, a S100A4 blocking antibody reduced the overall number of intratumor CD3+ T cells while increasing the number of intratumor CD209+ dendritic cells (117). There was a corresponding decrease in circulatory type 2 cytokines, including IL-4, IL-5, IL-6, and IL-13, as well as CCL5, suggesting that S100A4 blockade reverses the Th1/Th2 imbalance of CD4 T cells that occurs in these tumors (117). These findings align with S100A4’s purported role in altering the Th1/Th2 ratio by inhibiting Th1 polarization (**Figure 4a**).

In summary, S100A4 is a promising target in cancer therapy, as depletion of S100A4 reprograms the TME to a more anti-tumor environment. However, molecular mechanisms of S100A4 function in these aforementioned settings need further elucidation. Furthermore, additional studies are necessary to determine how and when targeting S100A4 in cancer synergizes with current, standard-of-care chemo, radiation, targeted, or immunotherapies such as anti-PD-1, anti-PD-L1, and anti-CTLA4, or CAR-T and other cellular therapies.

## 5 S100A4 inhibitors

Given its major role in promoting the progression of cancer, fibrosis, and chronic inflammatory conditions, there are ongoing efforts to develop inhibitors to target S100A4 signaling. Multiple S100A4 blocking antibodies have been reported and evaluated in preclinical models of breast cancer, prostate cancer, HCC, CRC, and skin fibrosis (49, 52, 65, 90, 117, 121). Treatment using the 6B12 antibody in mice decreased metastatic burden but did not change primary tumor size in the CSML100 breast cancer model (121). In the MMTV-PyMT spontaneous metastatic breast cancer mouse model, 6B12 and S100A4-11 antibody treatments reduced both number of metastases and metastatic burden at endpoint (90, 108). Within the premetastatic niche, 6B12 treatment reduced fibronectin deposition, g-CSF production, and T cell recruitment (90). In 4T1 breast model, S100A4-11 antibody also significantly reduced lung metastasis by inhibiting neutrophil infiltration and TIGIT signaling to NK and T cells in the lung to form premetastatic niche (108). Similarly, in a syngeneic mouse prostate cancer model, 6B12 treatment reduced the rate of tumor growth, indicating antibody drugs that target S100A4 hold great promise (117).

While there is no FDA-approved anti-S100A4 antibody therapy, a Phase 1 clinical trial has been successfully completed (NCT05965089). The 6B12 antibody and its humanized counterpart AX-202/CAL101 has also been tested in mouse models of skin fibrosis as well as human systemic sclerosis (SSc) patient skin samples (49, 51). 6B12 treatment showed decreased dermal thickness, myofibroblast count, and collagen deposition in a mouse model of fibrosis. Bulk RNAseq analysis of skin from control and treated animals showed that 6B12 antibody treatment resulted in downregulation of genes associated with inflammation as well as T cell chemotaxis and activation. These results suggest a reduction in inflammatory processes that characterize skin fibrosis. In human disease models, SSc patient derived fibroblasts treated with AX-202/CAL101 exhibited reduced profibrotic gene and protein expression compared to controls (122). Similarly, treatment of human SSc patient precision cut skin samples with AX-202/CAL101 resulted in downregulation of proinflammatory and profibrotic transcriptional pathways as measured by bulk RNAseq (51). Recently, a phase 1 study assessing AX-202/CAL101 in psoriasis patients (NCT05965089) was completed, which showed favorable safety profile. A phase 2 study in patients with IPF will begin recruitment in 2025 (press release from Calluna Pharma).

In the context of allergic disease, 6B12 antibody was also tested in mouse models of asthma and in PBMCs from human allergic rhinitis patients (11, 14). In OVA-sensitized and challenged mice, 6B12 treatment reduced airway hyperresponsiveness, inflammatory cell infiltrates, and release of inflammatory cytokines in bronchioalveolar fluid (11). In PBMCs from allergic rhinitis patients, 6B12 treatment reduced secretion of IL-5 and IL-13, a key cytokine involved in the pathogenesis of allergic rhinitis (14).

Niclosamide and trifluoperazine (TFP) have been identified as non-specific small molecule inhibitors of S100A4 through drug screens of luciferase reporters and S100A4/myosin IIA interaction, respectively (22, 43, 123–129). Niclosamide is an FDA-approved anti-helminthic and has been studied for repurposing and preclinical validation for different diseases such as fibrosis, RA, and asthma due to its anti-inflammatory properties (130, 131). As a possible S100A4-targeting therapeutic, niclosamide was first identified through a high-throughput screen with HCT116 human CRC cell line using a S100A4-promoter-driven luciferase reporter assay (125). Mechanistically, niclosamide inhibits an upstream regulator of S100A4 transcription, the Wnt signaling transcription effector complex CTNNB1/TCF complex (125). However, it is unclear how much of these changes can be attributed to S100A4 inhibition directly and how much other Wnt signaling targets. There are currently several clinical trials investigating niclosamide safety and efficacy in the treatment of cancer (132).

TFP is another FDA-approved, first-generation, anti-psychotic and a member of the phenothiazine family. TFP was identified through a biosensor-based assay as an inhibitor of S100A4/myosin-IIA interaction (133). Compared with other phenothiazines, TFP showed the highest ability to block S100A4 mediated depolymerization of myosin-IIA filaments (22). A crystal structure of S100A4 bound to TFP indicated that TFP disrupts the interaction between S100A4 and myosin-IIA by sequestering S100A4 into S100A4/TFP oligomers (22). TFP has been investigated as an anti-cancer therapeutic because of its function as a calmodulin (CaM) modulator, preventing calcium binding to CaM and increasing cytosolic calcium levels (134, 135). *In vitro* TFP treatment shows efficacy against human triple-negative breast cancer and glioblastoma (GBM) cell lines. *In vivo*, TFP suppresses tumor growth in GBM xenograft models (123, 134, 135), though it is unclear whether this is associated with its modulation of S100A4 function. TFP also has immune modulatory function, inhibiting pro-inflammatory cytokine release *in vitro* and *in vivo* in the context of sepsis (136). Despite these findings, there have been no further studies assessing TFP in therapeutic targeting of S100A4.

Given S100A4’s diverse function both intracellularly and extracellularly, antibody treatment likely provides a safe way to block S100A4 extracellular signaling to its receptors TLR4 and RAGE, but this will not affect intracellular function of S100A4. In contrast, small molecule inhibitors can likely target both intracellular and extracellular S100A4 function but lack the specificity of antibody treatments. Thus, rational strategies based on S100A4 biology in various contexts are necessary when deciding how best to target S100A4.

## 6 Concluding remarks

Review of the recent literature shows that S100A4 function in the immune system plays several key roles in the progression of cancer, fibrosis, and autoimmune diseases (**Table 1**). While much has been revealed of its importance in macrophages, T cells, MDSCs, and DCs, the molecular mechanisms through which S100A4 acts in each cell type is largely unknown. Given its ability to function both extracellularly as a DAMP and cytokine, as well as intracellularly in both the cytoplasm and at the nucleus, cell type specific and mechanistic studies are necessary to determine the specific mechanisms in different diseases.

**Table 1:** Function of S100A4 in immune cells.

Immune Cell Type	Disease Context	Mechanism	Perturbation	Reference
Macrophages	Peritonitis	Deficient CSF1R signaling, over-assembly of Myosin-IIA	Genetic Knockout	(23)
Macrophages/TAMs	Breast Cancer	S100A4 expression regulates induction of M2-like state through PPAR-γ signaling, with upregulation of immunosuppressive markers CD206, Arg1, and TGF-β	Genetic Knockout	(36)
Macrophages/TAMs	Glioma	Loss of S100A4 increases phagocytic ability of tumor infiltrating macrophages in mouse models of glioma	Genetic Knockout	(15)
Macrophages/Alveolar Macrophages	Lung Fibrosis	IL-4 polarization of macrophages drives S100A4-dependent activation of lung fibroblasts	Genetic Knockout	(43)
Macrophages	Lung Fibrosis	Adoptive transfer of WT macrophages reverses protection against bleomycin-induced lung fibrosis in *S100a4 -/-* mice	Genetic Knockout, Adoptive Transfer	(50)
RAW 264.7	CRC	S100A4 activates NF-κβ signaling through RAGE to promote inflammation	Extracellular treatment, RAGE inhibitor	(35)
THP-1	Breast Cancer	S100A4 polarizes THP-1 cells to promote mesenchymal transition of breast cancer cells	Extracellular treatment, Genetic Knockout	(42)
PBMCs	RA	S100A4 enhances inflammatory response of PBMCs from patients with RA through TLR4 signaling	Extracellular treatment	(9)
MDSCs	Cancer	S100A4 activates TLR4-ERK1/2 signaling axis to protect MDSCs from apoptosis to promote tumor progression	Genetic Knockout	(58)
MDSCs	AML	S100A4 binds to GP130 to activate JAK2/STAT3 signaling axis to promote MDSC expansion and T cell suppression	Overexpression, antibody treatment, knockdown	(77)
MDSCs	Sjogren’s syndrome	Exosome-mediated S100A4 signaling in MDSCs promotes IL-6 production via TLR4 activation, leading to downstream activation of JAK2/STAT3 signaling	Extracellular treatment	(29)
DCs	Allergic dermatitis	Endogenous S100A4 expression and extracellular S100A4 treatment in DCs promotes T cell activation	Genetic Knockout, extracellular treatment	(14, 81)
DCs	Autoimmune pancreatitis	TGFβR2 signaling mediates tolerogenic function in S100A4 expressing DCs	Genetic Knockout	(85)
T cells	Breast Cancer Lung Metastasis	S100A4 secretion from fibroblasts recruits CD4 T cells to pulmonary premetastatic niches to promote metastasis in the MMTV-PyMT spontaneous metastatic breast cancer mouse model; extracellular S100A4 prevents Th1 polarization through JAK/STAT activation	Extracellular treatment, Genetic Knockout, Antibody blockade	(90, 91)
CD8 T cells	Allergic Asthma	S100A4 expression upregulates mitochondrial metabolism, depleting Acetyl-CoA levels to reduce Ifng effector gene transcription and downstream effector function to promote allergic asthma	Genetic Knockout, Adoptive transfer, Extracellular treatment	(12)
CTLL2 cells	Liver Fibrosis	Extracellular S100A4 signaling activates the Akt pathway to prevent apoptosis	Antibody blockade, Akt inhibitor	(65)
Jurkat cells	RA	S100A4 regulates CD5 activity to promote RORγT expression and Th17 polarization in RA	Genetic Knockout	(87)

TAMs, Tumor-associated macrophages; RA, Rheumatoid arthritis; PBMCs, Peripheral blood mononuclear cells; MDSCs, Myeloid derived suppressor cells; DCs, Dendritic cells; AML, Acute myeloid leukemia.

S100A4 was initially nominated as a therapeutic target in the context of cancer metastasis. The field has since evolved in its understanding of how S100A4 promotes multiple aspects of cancer progression through immune modulation and cancer cell aggressiveness. In addition, S100A4 has emerged as a therapeutic target in fibrosis, again as an immune modulator. S100A4 involvement in these pathological processes is intimately intertwined with its role in regulating systemic and local immune response. Of particular importance is distinguishing the extracellular and intracellular functions of S100A4, as the former is much more readily targetable via antibody blocking, while the latter may require small molecule inhibitors or engineered biologics. Notably, *S100a4 -/-* mice are viable and fertile, indicating that it is a non-essential gene and potential side effects anticipated from S100A4 inhibitors is minimal. In line with this, the Phase1 trial by Calluna reported favorable safety profile and no serious adverse events. In addition, it will be important to target S100A4 in a cell-type and context-dependent manner, given its differing effects between immune cells and in different diseases.

Crucially, many knowledge gaps remain in our mechanistic understanding of how S100A4 expression and secretion in various cell types promote disease states. Most prior *in vivo* studies regarding S100A4 function have been performed with germline knockout of *S100a4* in mice. It is difficult to delineate which of the interpreted *in vivo* phenotypes are a direct result of S100A4 function in specific immune cells or are secondary effects of S100A4 deficiency in another immune or stromal cell, including fibroblasts and mesenchymal cells. Using conditional deletion with lineages specific Cre-mice in combination with single-cell methodologies can help to resolve these questions. In addition, while we know that both intracellular and extracellular S100A4 play important roles in these processes, little is known about which specific S100A4 targets, receptors, and downstream signaling pathways mediate these processes in different disease states and cell types. It will be critical to elucidate which S100A4 receptors regulate different immune cell function in various disease contexts, allowing for more targeted approaches for disease modulation.

In conclusion, unraveling the complex interactions between S100A4 and the immune system can open new avenues for therapeutic interventions in cancer, fibrosis, and pro-inflammatory conditions.
